# A quality improvement program in pediatric practices to increase tailored injury prevention counseling and assess self-reported changes made by families

**DOI:** 10.1186/s40621-018-0145-z

**Published:** 2018-04-10

**Authors:** Michael A. Gittelman, Adam C. Carle, Sarah Denny, Samantha Anzeljc, Melissa Wervey Arnold

**Affiliations:** 1 Division of Emergency Medicine, Cincinnati Children’s Hospital Medical Center, College of Medicine, University of Cincinnati, 3333 Burnet Avenue, MLC 2008, Cincinnati, OH 45229 USA; 2 James M Anderson Ctr for Health Systems Excellence, Cincinnati Children’s Hospital Medical Center, 3333 Burnet Avenue, Cincinnati, OH 45255 USA; 3 Division of Emergency Medicine, Nationwide Children’s Hospital, 700 Childrens Dr, Columbus, OH 43205 USA; 4 Ohio Chapter of the AAP, 94-A Northwoods Blvd., Columbus, Ohio 43235 USA

**Keywords:** Injury prevention, Quality improvement, Pediatric office visit, Behavior change

## Abstract

**Background:**

Many pediatric providers struggle to screen families for the majority of age-appropriate injury risks and educate them when appropriate. Standardized tools have helped physicians provide effective, more purposeful counseling. In this study, pediatricians utilized a standardized, injury prevention screening tool to increase targeted discussions and families were re-screened at subsequent visits to determine changes in their behavior.

**Methods:**

Pediatric practices, recruited from the Ohio Chapter, American Academy of Pediatrics database, self-selected to participate in a quality improvement program. Two screening tools, for children birth-4 month and 6–12 month, with corresponding talking points, were to be implemented into every well child visit. During the 7-month collaborative, screening results and pediatrician counseling for reported unsafe behaviors were calculated. Patients who completed a screening tool at subsequent visits were followed up at a later visit to determine self-reported behavior changes. We examined statistically significant differences in frequencies using the *X*^2^ test. Providers received maintenance of certification IV credit for participation.

**Results:**

Seven practices (39 providers) participated. By the second month, participating providers discussed 75% of all inappropriate responses for birth-4 month screenings and 87% for 6–12 months. Of the 386 families who received specific counseling and had a follow-up visit, 65% (*n* = 94/144) of birth-4 month and 65% (*n* = 59/91) of 6–12 month families made at least one behavior change. The X^2^ test showed that families who received counseling versus those that did not were significantly more likely to change inappropriate behaviors (*p* < 0.05). Overall, of all the risks identified, 45% (136) of birth-4 month and 42% (91) of 6–12 month behaviors reportedly changed after a practitioner addressed the topic area.

**Conclusions:**

Participation in a quality improvement program within pediatric offices can increase screening for injury risks and encourage tailored injury prevention discussions during an office encounter. As a result, significantly more families reported to practice safer behaviors at later visits.

## Background

Injuries continue to cause significant morbidity and mortality to US children. Unfortunately, close to 20 children die daily from a preventable injury; causing more deaths than all diseases combined (Sleet et al., 2002). As deaths from infectious diseases and chronic conditions have declined over the past century, the proportion of pediatric deaths attributable to unintentional injuries remains stable (Johnson et al., 2014). Deaths are merely the tip of the iceberg. Injured children account for more than 2 million outpatient visits, 9 million emergency department (ED) encounters and 225,000 hospital admissions annually; ultimately, costing society over $87 billion each year (Anderson et al., 2010; Centers for Disease Control and Prevention. National Hospital Ambulatory Medical Care Survey, 2010). Many high-income countries have been able to reduce their child injury deaths by up to 50% over the past three decades by implementing multi-pronged preventive efforts (Harvey et al., 2009).

Professional societies and national task forces suggest that all primary care providers (PCPs) counsel families about age-appropriate injury risks at well-child visits (WCV) to help prevent future injuries (Committee on Injury, Violence, and Poison Prevention, American Academy of Pediatrics, 2007; American College of Surgeons, 2013; US Preventative Services Task Force, 2013). Despite recommendations, PCPs struggle to provide these discussions comprehensively at every encounter (Gielen et al., 1997; Grossman & Rivara, 1992; Grossman et al., 1995). Challenges such as a lack of time, too many topics to cover, and a lack of training about injury prevention (IP) issues have been cited as barriers (Yarnall, 2003; Belamarich et al., 2006; Wright, 1997).

Injury prevention screening tools were first introduced by the American Academy of Pediatrics (AAP) when they developed their Framingham Safety Surveys, a part of the The Injury Prevention Program (TIPP)® (Krassner, 1984). This type of tailored IP screening, with individualized, custom messaging, helps to address specific needs and can entice greater behavioral change than population-based, generic handouts (Nansel et al., 2002). By implementing these tools into pediatric offices using quality improvement (QI) methodology, PCPs are able to screen for and discuss high-risk injury topics in a more pertinent and efficient manner (Gittelman et al., 2015).

When the same IP screening tool is used on multiple encounters at WCVs, changes in subsequent responses and risky behaviors can be assessed. The purpose of this study was to determine the self-reported behavior changes on injury screens at repeated visits during a pediatric office-based QI program.

## Methods

### Study design

We conducted a prospective study of family safety behaviors evaluating pre- and post- targeted pediatric provider IP counseling at WCVs. The data for this study were collected during our second wave of a Quality Improvement Learning Collaborative (QILC) conducted by the Ohio Chapter, American Academy of Pediatrics (OAAP) from October 18, 2013 to May 31, 2014 (Gittelman et al., 2015). The aim of the collaborative was to assist PCPs on screening families, with children ≤1 year of age, for injury risk and counseling them about appropriate behavior changes. The primary objective of the research was to evaluate self-reported behavior changes by families on subsequent office visits, based on parental screens after PCP’s offered recommendations. The main objective of the QILC was to have pediatricians address > 90% of injury behaviors in which families screened at risk in the office setting.

### Setting and QI program

The QILC structure was similar to the Institute of Healthcare Improvement (IHI) Breakthrough Series Collaborative (Institute for Health Care Improvement (IHI), 2003). Practitioners were recruited from the OAAP membership database and volunteered to participate. Mailings and postings in newsletters were sent to members notifying them of our Maintenance of Certification IV opportunity. Study teams were chosen by each participating practice and consisted of a physician leader, a nurse/nurse practitioner or medical assistant, and an administrative staff/office manager. Core teams participated in a pre-work conference call outlining the requirements for the QILC and the collection of baseline data (a three-month retrospective chart review of 36 randomly selected WCV charts of children ≤1 year of age to evaluate the IP anticipatory guidance (AG) discussions and documentation that occurred).

After collecting baseline data, all core team members from each practice attended a one-day face-to-face learning session held on October 18, 2013. Learning session objectives were to educate team members about the importance of discussing IP at a WCV, principles of QI methodology, including how to conduct Plan-Do-Study-Act (PDSA) cycles, how to implement the IP screening tool into practice, and how monthly data should be collected and reported. We condensed our previously developed six IP screening tools into two tools for this QI program (birth to 4 month and 6 month to 1 year screens) (Gittelman et al., 2015). All topics and questions were based on age-appropriate IP recommendations adapted from the AAPs TIPP program,^®^ and they were reviewed by experts from the AAP’s Council on Injury, Violence, and Poison Prevention (Patient Education Online, 2017). A test-retest study has shown that these questions have good reliability (Gittelman et al., 2016). Each tool addressed four to six injury topics with several questions per topic (total of approximately 20 questions per survey). Questions concentrated on current behaviors of families more than attitudes about prevention. On average, surveys took approximately 5–7 min to complete by families. At the conclusion of the learning session, each team was provided with both forms of the IP screening tool, grading sheets, and physician talking points.

During the QILC, teams worked to employ the developed IP screening tools within their practice for every WCV, ill visits were not included, and PCPs tried to address all risky behaviors elicited from families. Each month, core team members participated in a conference call and webinar to review identifiable practice level and collaborative data, with no patient personal health information (PHI), to foster peer-to-peer discussions, determine areas of success, and needs for improvement. In addition, these calls always consisted of a 15-min lecture on an injury topic relevant to children 1 year of age and younger. Finally, teams completed a monthly report outlining their office changes and PDSA cycles attempted.

All physician members in each practice that submitted data received American Board of Pediatrics (ABP) Maintenance of Certification (MOC) IV credit for participation.

### Data collection

#### Baseline

Participating pediatric providers reviewed 36 randomly selected charts (6 for each WCV: newborn, 2-month, 4-month, 6-month, 9-month and 12 month) from the previous 3 months of WCVs. A standardized protocol for chart review was provided to pediatric providers prior to the learning collaborative and reviews were entered into the OAAP’s QI dataspace by staff from each practice. De-identified patient-level responses were entered; no PHI was included within the OAAP database. Practices received their baseline reports at the one-day learning collaborative.

#### Action period

After the one-day collaborative, each month pediatric providers reviewed all WCV charts for children ≤1 year. Similar to baseline, all chart reviews were entered into the OAAP QI dataspace. PHI was only visible to participating providers and used to match primary screens with secondary visits. The OAAP dataspace only contained de-identified patient-level data, as pre and post tool matching was done by the participating practices. Thus, the OAAP dataspace did not require HIPPA protection. This aggregate data were secured and only analyzed by the study staff. Topics were considered addressed if the family answered the screening question appropriately (based on the provided answer key) or if the provider checked the discussed box for those questions answered inappropriately. Frequencies were determined to assess all age appropriate topics addressed by the PCP at WCVs. Changes in providers addressing risky topics over time was determined and presented individually and in aggregate on monthly action period calls. This provider level data were not blinded during these calls in order to increase competition and to help share successes and areas for improvement. When addressing appropriate and inappropriate responses at repeat screens, previous screening was not provided to the PCP to prevent bias from pediatric provider counseling.

### Analysis

Families who completed the same age grouped screening tool on subsequent visits (pre and post screenings) were followed up at a later visit to determine self-reported behavior changes after discussion was provided by the PCP. Repeat screening tools completed by a different caregiver were omitted from the behavior change analyses due to the lack of reliability when the tool is used with different caregivers (Gittelman et al., 2016). We examined statistically significant differences in frequencies using a *X*^2^ test.

### Human subjects review

Approval was obtained from the Cincinnati Children’s Hospital Institutional Review Board prior to study initiation.

## Results

During the action period, seven practices with 39 pediatric providers participated and remained active in the QILC. No practitioner dropped out of the QILC after collection of baseline data. Practices varied by size, number of patients seen annually, setting and population served (Table 1). In one practice, residents participated along with attending physicians. In this practice, the tool was implemented and tailored discussions were completed similarly as the other practices.

**Table 1:** Participating practice characteristics

Practice	Number of Providers	Estimate of Annual Patient Volume - Birth to 1 Year	Practice Setting	% Medicaid	% Private Insurance	% Self-Pay
1	6	3000	Suburban	30	70	0
2	7	2820	Suburban	10	80	10
3	1	396	Urban	90	0	10
4	10	396	Suburban	0	90	10
5	6	1680	Rural	100	0	0
6	2	252	Urban	80	10	10
7	25 Residents & 7 Attending	468	Urban	40	50	10

There were 1858 initial screens completed during the action period of the QILC. 56% (1049) were for birth-4 month olds and 44% (809) were for 6–12 month olds. There were 20.8% (386) repeat, follow-up screens completed. Of the follow-up screens, 53% (205) were for 0–4 month olds and 47% (181) were for 6–12 month olds. In general, respondents answered the majority of screening questions appropriately. On average, families answered only 1.44 of the birth-4 month questions inappropriately, while 1.46 of the 6–12 month questions were answered incorrectly. Table 2 depicts the most common questions answered appropriately and inappropriately by families.

**Table 2:** Top 5 questions answered appropriately, inappropriately, and not discussed by PCPs at the initial screening encounter

Top 5 Question Answered Appropriately	N-4 Month (*n* = 1049)			6–12 Month (*n* = 809)		
	Question	% Appropriate		Question	% Appropriate	
	No Sitters < 12 years old	99.81%		Not in Yard when Yard Equipment is in Use	99.25%	
	Does not leave child alone in tub	99.81%		Vitamins and meds are properly stored	99.12	
	Does not leave child alone in pool	99.81%		Rides in a car seat	97.65%	
	Does not witness physical expressions of anger when frustrated	99.62%		Does not consider leaving child alone in car	97.27%	
	Not afraid of significant other	99.5%		Harmful cleaners/pesticides away from child	96.87%	
Top 5 Questions Answered Inappropriately	N-4 Month (n = 1049)			6–12 Month (n = 809)		
	Question	% Appropriate		Question	% Appropriate	
	Car Seat Checked by Professional	47.66%		Not likely to get Small Objects	40.2%	
	Following Safe Sleep Practices*	48.37%		CPR course in Past 3 Years	48.49%	
	Working CO Detector	61.28%		Car Seat Checked by Professional	52.11%	
	Working Fire Extinguisher	62.33%		Furniture is attached to walls	52.65%	
	Hot Water Heater set to < 120	64.34%		Poison Control # Clearly Posted	59.5%	
*Families answering appropriately to all three sleep questions.						
Top 5 Topics not Discussed	N-4 Month (n = 1049)			6–12 Month (n = 809)		
	Question	# answered incorrectly	% Addressed	Question	# answered incorrectly	% Addressed
	Collective discussion of safe sleep topics (shared space, on back, no bumpers, no objects)	711	22%	Poison Control # Clearly Posted	433	31%
	Car Seat Checked by Professional	663	21%	CPR course in Past 3 Yrs	567	29%
	Working CO Detector	399	18%	Furniture is attached to walls	503	25%
	Working Fire Extinguisher	469	17%	Has Window Guards	400	25%
	Smoke Alarm batteries changed in past 6 mo.	186	14%	Not likely to get Small Objects	606	17%

Prior to the start of the QILC, no practice utilized any type of IP screening tool. After the second month of the action period, participating PCPs discussed 75% of all inappropriate responses on completed screens for birth-4 month screenings and 87% for 6–12 month screenings. This frequency was continued for the remainder of the QILC; demonstrating early maintenance and sustainability of improvement efforts. The most common reasons for consistency cited by practices included: a consistent site for tool distribution (most commonly the waiting room area), a designated person accountable for scoring tools, and the perceived efficiency providers felt about their AG discussions.

Of the 386 children for whom repeated screens were completed, the same caregiver completed 88%, for both the birth-4 month (249/282) and 6–12 month (92/104) screenings, at the child’s next age-consistent follow-up visit. When comparing their initial visit to follow-up, a substantial number of families made at least one change in their behavior after the PCP made recommendations - birth to 4 months 65% (93/144) and 6 months to 1 year 65% (59/91). Among children with follow-up visits, 45% (136) of inappropriate responses made by families with children birth to 4 months were corrected by their follow-up visit if the PCP addressed the risk with the family. Similarly, 42% (91) of inappropriate responses made by 6 month to 1-year families were corrected by their follow-up visit if the PCP addressed the risk with the family. Families also, occasionally, made changes in their behavior even if the PCP failed to address all inappropriate responses. Thirty-eight percent (106) of birth to 4 month and 26% (33) of 6 to 12 month responses were corrected even though the PCPs did not address the behavior at the office visit.

Overall (with or without a discussion), 42% of birth to 4 months and 36% of 6 to 12 months of all inappropriate behaviors were corrected after their WCV as determined on subsequent screening. Table 3 presents the number of changed and unchanged behaviors that occurred when 1) physicians discussed inappropriate behaviors and 2) physicians did not discuss inappropriate behaviors. Looking at Tables 2 and 3 together, one can see that, for both age groups, the more common an inappropriate behavior was (e.g. not having a working fire extinguisher: Table 2) the less likely it was to be corrected at follow-up in general.

**Table 3:** Changed and unchanged behaviors reported based on discussions with PCP

		Behavior Discussed				Behavior Not Discussed			
		Number Unchanged	Number Changed	Total Inappropriate Behaviors	% Changed	Number Unchanged	Number Changed	Total Inappropriate Behaviors	% Changed
0–4 Month Olds	Convertible car seat?	3	5	8	62.50	1	3	4	75.00
	Seat direction?	1	4	5	80.00	0	1	1	100.00
	Car seat checked?	3	3	6	50.00	86	11	97	11.34
	Consider leaving in car?	0	4	4	100.00	–	–	–	–
	Sleep in shared space?	29	18	47	38.30	9	6	15	40.00
	Sleep on back?	–	–	–	–	10	12	22	54.55
	Sleep with bumpers?	–	–	–	–	23	23	46	50.00
	Sleep with pillows?	–	–	–	–	17	19	36	52.78
	Sleep with objects?	–	–	–	–	1	5	6	83.33
	Sleep sack?	4	5	9	55.56	2	4	6	66.67
	Smoke alarm?	2	2	4	50.00	0	2	2	100.00
	Alarm batteries changed?	7	16	23	69.57	1	2	3	66.67
	Water temp?	39	35	74	47.30	9	8	17	47.06
	Working fire extinguisher?	46	15	61	24.59	10	4	14	28.57
	Hot liquids?	1	4	5	80.00	1	1	2	50.00
	Carbon monoxide detector?	30	20	50	40.00	5	4	9	44.44
	Young than 12 watching?	–	–	–	–	–	–	–	–
	Feel afraid of partner?	–	–	–	–	–	–	–	–
	Verbal anger?	0	2	2	100.00	–	–	–	–
	Physical anger?	–	–	–	–	0	1	1	100.00
	Alone on surface?	1	3	4	75.00	–	–	–	–
	Alone in tub?	–	–	–	–	–	–	–	–
	Alone in pool?	–	–	–	–	–	–	–	–
6–12 Month Olds	How often convertible?	0	3	3	100.00	0	–	–	–
	Seat direction?	2	3	5	60.00	0	–	–	–
	Car seat checked?	1	1	2	50.00	34	11	45	24.44
	Consider leaving in car?	0	3	3	100.00	0	1	1	100.00
	Have barrier?	6	9	15	60.00	0	3	3	100.00
	Window guards?	13	17	30	56.67	10	4	14	28.57
	Water fence?	1	0	1	0.00	0	–	–	–
	Small objects into hands?	36	12	48	25.00	8	2	10	20.00
	Non-mashable foods?	5	2	7	28.57	0	–	–	–
	Lifesaving techniques?	27	4	31	12.90	27	4	31	12.90
	Around lawn equipment?	–	–	–	.	0	–	–	–
	Secured furniture?	18	16	34	47.06	10	4	14	28.57
	Locked guns?	–	–	–	.	–	–	–	–
	Locked meds?	–	–	–	.	–	–	–	–
	Locked cleaners?	1	3	4	75.00	0	1	1	100.00
	Poison control number?	16	18	34	52.94	4	3	7	42.86

Cells with no values indicate either that no PCP discussed the behavior (even if a parent reported inappropriate behaviors), that all PCPs discussed the behavior, or that no parents reported an inappropriate behavior for a given question

## Discussion

This study demonstrates several important findings regarding the ability to screen and discuss IP risks in the pediatric office setting and the effectiveness of this counseling. Unfortunately, PCPs are still infrequently discussing IP at primary care visits (Hammig & Jozkowski, 2015). Previous studies have shown that practitioners can easily implement an IP screening tool into routine WCVs to increase the number of IP topics covered (Gittelman et al., 2015). However, this study has shown that using QI methodology can substantially increase practitioner’s discussions of inappropriate behaviors with families. This behavior can be changed in a relatively short time and it can be maintained at consistent levels. When families return for subsequent WCVs, families are significantly more likely to report (through pre and post-screening) that they made positive changes in their behavior after receiving PCP recommendations.

The primary QI procedure for each provider was to implement our injury-screening tools into practice and discuss uncovered risks. Integration of the screening tool into office flow was relatively quick, within 2-months after the learning session, and sustained throughout the QILC. Despite the goal to have PCPs discuss all inappropriate responses at the initial visit, PCPs were able to address at least 80% of behavior discussions at the initial visit. The most common reasons for not attaining 100% on injury discussions cited by participants were: lack of time and uncertainty of the appropriate message. These reasons for limited injury prevention discussions are similar to those listed in other studies (Yarnall, 2003; Belamarich et al., 2006; Wright, 1997). However, on follow-up surveys of participating PCPs, they stated that they felt using the provided tool made them more efficient with injury prevention discussions and that the talking points helped them start the conversations more easily. This finding is consistent with other studies that used standardized injury screening tools in the office setting to encourage tailored discussions when appropriate risks are identified (Nansel et al., 2008). In addition, 1 year after completing the MOC program, PCPs reported that 60% were still using the injury screening tool and talking points on a randomized follow-up survey.

Several injury prevention risks were consistently identified when families were screened in the office. Approximately 48% of families reported their newborn to 4-month old did not follow recommended safe sleep practices: they slept with pillows or blankets, in a crib with bumpers, or in a shared space. Similarly, 53% had not had their car seat checked by a professional, while 38% did not have a working fire extinguisher, and 36% had their water heater greater than 120 degrees. For families with children 6 months to 1 year, approximately 60% had small objects easily accessible to their child, 48% did not have their car seat checked by a professional, and 47% did not have furniture securely attached. Implementing our injury-screening tool into daily practice, these specific child injury risks were identified. This enabled pediatricians to concentrate on particular risks instead of spending added time questioning families about all recommended age-appropriate IP topics. Ultimately, pediatric providers can be more efficient during their office time using this tool as they address injuries, the number one cause of death and disability for children.

The primary goal of this study, to determine if families self-reported behavior changes after risk was assessed and discussion occurred with their PCP, was shown to be significant. For both newborn-4 month and 6–12 month families, 65% reported a behavior change in at least one of their screened risky behaviors. Given the screening tool we used has demonstrated good test-retest reliability, it is likely that the reported behavior change we observed was valid (Gittelman et al., 2016). Also, families tended to make further behavior changes even if PCPs did not provide recommendations at their WCV. The change that occurred without a physician’s discussion is likely a “testing effect” (merely being asked about the behavior resulted in a change). However, our results suggest that not all of the change reflected a testing effect because positive change was significantly greater among families whose physician discussed inappropriate behavior than among those whose physician did not discuss in appropriate behavior.

These findings of behavior change are similar to other studies showing that repetitive and individualized safety education can improve parental safety practices (Kelly et al., 1987). In fact, in reviewing the literature on childhood injury-prevention counseling in the primary care setting, 18 of 20 studies have shown positive outcomes in increasing knowledge and behavior and in decreasing injury rates in children (Bass et al., 1993). IP counseling by pediatricians has even shown a positive effect on medical costs in which $11 for counseling per child, for families with children aged 0–4, can generate $97 in benefits to society (Miller & Galbraith, 1995).

This study is not without some limitations. First, the responses by families to elicit behavior change were self-reported. Families may have had social desirability to respond the way they felt the PCP would have wanted them to respond. Also, despite past studies supporting the reliability of the screening tool’s questions, respondents may inappropriately state a change in their behavior due to exposure bias, as they were screened with the same tool at a previous office visit. However, the re-visit occurred approximately 2 months after the initial visit and memory of all specific responses from their previous screen is unlikely. Another limitation is that some families did not have a follow-up visit. Although discussing behaviors did not significantly predict attrition, it is possible that the patterns we observed differed among families without follow-up observations. In addition, the complete number of screens performed by each practitioner was not determined. We encouraged practices to screen all children ≤1 year seen by the participating PCP; however, we were not able capture this information during the collaborative. Lastly, PCPs had a vested interest during the QILC to show change as they were participating to receive MOC credit. However, the PCPs were evaluated on their ability to address risky behaviors assessed on screen, not the positive change on subsequent visits. In addition, PCPs did not have immediate access to the families’ initial screens so these responses were not known at the second visit.

## Conclusions

This study shows that PCPs can utilize a standardized IP screening tool consistently, with sustained improvement efforts in their office practice. The tool enables providers to screen for more age-appropriate injury risks and thus counsel about more topics. When guardians are screened on subsequent encounters, a large proportion of them report to have made a behavior change, making their child safer from future injury. Future studies need to validate these findings by observing family behaviors in the home setting compared to their responses on screen. Other studies could also include if behavior changes by pediatricians and families are sustained over time, if similar behavior changes would be made if other providers in the office provided counseling besides the pediatrician and if these results could be duplicated in other settings such as family practice offices.

## Abbreviations

AAP: American Academy of Pediatrics
ABP: American Board of Pediatrics
AG: Anticipatory guidance
ED: Emergency department
IHI: Institute of Healthcare Improvement
IP: Injury prevention
MOC: Maintenance of Certification
OAAP: Ohio Chapter, American Academy of Pediatrics
PCP: Primary care provider
PDSA: Plan-Do-Study-Act
PHI: Personal health information
QI: Quality improvement
QILC: Quality improvement learning collaborative
TIPP: The Injury Prevention Program^®^
WCV: Well child visit
